# Kynurenines and Multiple Sclerosis: The Dialogue between the Immune System and the Central Nervous System

**DOI:** 10.3390/ijms160818270

**Published:** 2015-08-06

**Authors:** Cecilia Rajda, Zsófia Majláth, Dániel Pukoli, László Vécsei

**Affiliations:** 1 Department of Neurology, University of Szeged, Szeged H-6725, Hungary; E-Mails: rajda.cecilia@med.u-szeged.hu (C.R.); majlathzsofia@gmail.com (Z.M.); pukoli.daniel@med.u-szeged.hu (D.P.); 2 Department of Neurology, Markhot Ferenc County Hospital, Eger H-3300, Hungary; 3 MTA-SZTE Neuroscience Research Group, Szeged H-6725, Hungary

**Keywords:** kynurenine pathway, multiple sclerosis, indoleamine-2,3-dioxygenase

## Abstract

Multiple sclerosis is an inflammatory disease of the central nervous system, in which axonal transection takes place in parallel with acute inflammation to various, individual extents. The importance of the kynurenine pathway in the physiological functions and pathological processes of the nervous system has been extensively investigated, but it has additionally been implicated as having a regulatory function in the immune system. Alterations in the kynurenine pathway have been described in both preclinical and clinical investigations of multiple sclerosis. These observations led to the identification of potential therapeutic targets in multiple sclerosis, such as synthetic tryptophan analogs, endogenous tryptophan metabolites (e.g., cinnabarinic acid), structural analogs (laquinimod, teriflunomid, leflunomid and tranilast), indoleamine-2,3-dioxygenase inhibitors (1MT and berberine) and kynurenine-3-monooxygenase inhibitors (nicotinylalanine and Ro 61-8048). The kynurenine pathway is a promising novel target via which to influence the immune system and to achieve neuroprotection, and further research is therefore needed with the aim of developing novel drugs for the treatment of multiple sclerosis and other autoimmune diseases.

## 1. Introduction

Multiple sclerosis (MS) is a chronic disease with mainly inflammatory features in the beginning and later neurodegenerative processes take over. There is an overlap of the pathological processes during the time course. Patients are considered to be clinically active if they have two relapses in two years and may have gadolinium enchancing lesion(s) on brain magnetic resonance imaging (MRI). Chronic patients have no relapses, their disease course is slowly progressing from the beginning (primary progressive course) or slowly progressing after a relapsing-remitting course (secondary progressive). It became more and more evident that the acute plaques in the brain differ from the chronic plaques regarding their underlying pathomechanism.

In this inflammatory disease of the central nervous system (CNS) axonal transection takes place in parallel with acute inflammation. The extents of the two major pathomechanisms are individual. The demyelination appears to be extensive also in the gray matter and global neuron degeneration is also observed. The inflammation affects the energy metabolism of the axons through the mitochondria, and local oedema influences the local blood flow causing ischemic axonal degeneration. Toxic mediators lead to breakdown of the blood-brain barrier and damage to the myelin sheath and axons. Those mediators are tumor necrosis factor-α (TNF-α), interferon-γ (IFN-γ), nitrogen-monoxid (NO) and reactive oxygen species.

The pathomechanism in MS is heterogeneous, but in a given individual the same pattern is present throughout the disease course. In the active inflammatory form, four subtypes have been described, which differ as regards the molecules taking part in the process: (1) T cell and macrophage-mediated; (2) B cell (antibody) and complement-mediated; (3) oligodendrocyte apoptosis-induced and (4) A primary oligodendrocyte apoptotic form.

In sequence of appearence, the most common form is 2, followed by 3, 1 and 4 [1].

The inflammation in MS may trigger a pathogenetic cascade of events leading to neurodegeneration, amplified by mechanisms related to brain aging and an accumulated disease burden. The factors causing this neurodegeneration include chronic oxidative injury, microglia activation, accumulation of mitochondrial damage in the axons and age-related iron accumulation in the brain. An altered mitochondrial function can cause neuronal death by the inbalance of ionic homeostasis and chronic cell stress [2]. The most characteristic lesion of MS is demyelination where the axons are partially preserved. However, in progressive MS brain atrophy is the most prominent pathological finding. In the course of relapsing-remitting MS (RRMS), actively demyelinating plaques associated with inflammation and blood-brain barrier injury are observed, but they are rare in the progressive course [2].

In chronic MS, the lesions shrink even in the presence of ongoing activity at the margins, contributing to brain atrophy. It is caused in part by the degeneration of the chronically demyelinated axons. Axonal degeneration begins in active MS lesions. Most axons survive the acute but not the chronic states of demyelination. In progressive MS, therefore, the degeneration of chronically demyelinated axons is a prominent feature, and is also a major cause of irreversible disability [2].

The blood-brain barrier damage observed with inflammation in the active white matter lesions in early MS differs in patients with progressive MS, in whom perivascular and parenchymal inflammation are seen, at least partly, in the absence of serum protein leakage from affected blood vessels. This means that such an inflammatory reaction is no longer reflected by contrast enhancement on MRI, and drugs that target this type of inflammation should be able to enter the CNS through an intact blood-brain barrier [2].

Another pathological feature of progressive MS is cortical demyelination, which is probably one of the causes of the cognitive disability suffered by the patients [3,4]. While early relapsing and remitting disease is associated with the appearance of focal plaques in the white matter, cortical demyelination, and diffuse alterations of the white and gray matter are present in the progressive stage [2].

## 2. The Kynurenine Pathway

The essential aminoacid tryptophan (Trp) can be metabolized in two main routes: the serotonin and the kynurenine pathways (KP) [5,6]. The serotonin pathway is better known, though more than 95% of the Trp is transformed through the KP [7]. The first and rate-limiting step of the KP, the enzymatic transformation of Trp into L-kynurenine (L-KYN), can be catalyzed by either tryptophan-2,3-dioxygenase (TDO) or indoleamine-2,3-dioxygenase (IDO). TDO is predominantly located in the liver cells, but in smaller amounts it can be found in the brain as well. IDO is present in numerous different cell types such as the microglia, neurones or astrocytes. At the level of L-KYN, the KP divides into three branches: it can be metabolized into kynurenic acid (KYNA), or it can produce anthranilic acid or it will be degraded in a sequence of enzymatic steps responsible for the formation of neurotoxic kynurenines. KYNA is produced by kynurenine-aminotransferases (KATs). The four known KAT isoforms have slightly different biochemical properties; KAT-II is mainly responsible for KYNA synthesis in the human brain. KYNA is the only known endogenous antagonist of ionotropic glutamate receptors, and it therefore has a significant neuroprotective property [8]. It is a competitive antagonist of *N*-methyl-d-aspartate (NMDA) receptors [9,10]. Interestingly, it has a dose-dependent dual effect on the α-amino-3-hydroxy-5-methyl-4-isoxazolepropionic acid (AMPA) receptors: in lower concentrations it evokes facilitation, while in higher amounts it has an inhibitory effect [11,12]. Another important effect of KYNA is the inhibiton of presynaptic α7 nicotinic acetylcholine receptors thereby regulating presynaptic glutamate release [13,14]. As even low concentrations of KYNA are able to inhibit glutamate release, this effect contributes significantly to its neuroprotective effect besides NMDA antagonism.

Another branch of the KP produces several neuroactive kynurenines which are mainly neurotoxic. 3-hydroxy-kynurenine (3HK) is formed from L-KYN through the action of kynurenine-3-monooxygenase (KMO). The third branch of the KP is the formation of anthranilic acid from L-KYN by the action of kynureninase. Anthranilic acid and 3HK are both converted into 3-hydroxy-anthranilic acid, which is degraded further and yields quinolinic acid (QUIN). 3HK and QUIN are both neurotoxic molecules, which in higher conentrations may result in neuronal damage. QUIN is a potent agonist of NMDA receptors, which may thereby induce glutamatergic excitotoxicity [15]. QUIN may also induce lipid peroxidation and oxidative stress [16]. 3HK and 3-hydroxy-anthranilic acid contribute to free radical production and therefore strengthen the neurotoxic effects of QUIN. The enzymatic machinery is differently distributed in the different cell types: the astrocytes harbor mainly KATs and hardly any KMO, while microglial cells have predominantly KMO and therefore produce mainly the neurotoxic kynurenines [17,18].

## 3. The Role of the KP in Immunoregulation

The importance of the KP in the physiological functions and pathological processes of the nervous system has been extensively investigated, and it has recently been implicated as having a regulatory function in the immune system too [19]. In this relation, IDO is of outstanding importance, and may take part in the immunoregulation through Trp depletion and the production of kynurenines. IDO is present in various immune cells, including monocytes, macrophages and microglia [20]. IDO can be induced by interferons and LPS too, but IFN-γ is considered to be the main activator. The activation of IDO results in an elevation of the levels of KYNA and other kynurenines, and it is considered to be an important regulator of immune activation as it counteracts the proliferation of reactive lymphocytes. This is partly a result of Trp depletion, while on the other hand, QUIN and 3-hydroxyanthranilic acid result in the selective apoptosis of TH1 cells [21]. This function is considered to play a significant role in the regulation of the immune response as a negative feedback loop and in the development of immune tolerance [22,23]. Importantly, IDO-mediated T-cell inhibition prefers Th1 cells, though, it may additionally counteract Th2 cells through the activation of regulatory T-cells [20].

## 4. The Role of the KP in the Pathomechanism of MS

### 4.1. Preclinical Results

Experimental autoimmune encephalitis (EAE) is a T-cell-mediated autoimmune animal model for MS that is histologically similar to human MS [24,25]. The EAE is until now the best animal model representing the relapsing-remitting form of the disease, however there are known shortcomes, e.g., the progressive phase is not modelled, *etc.* Kwidzinski *et al.* [26] demonstrated the importance of the immune modulating effect of IDO-1 in EAE. The inhibition of the enzyme activity significantly decreases the neuroinflammatory process, resulting in a decrease of the exacerbation of the disease. In the spinal cord and brainstem of rats with EAE, KMO immunoreactivity has been found in the cytoplasmic granules. Another neurotoxic KP metabolite, 3-HK, has been found to be increased in the spinal cords of rats with EAE [27]. Flanagan *et al.* [28] measured a selective QUIN level increase in the spinal cords of EAE rats. Both EAE models have been induced by the same methods, resulting in an acute clinical course. Cammer *et al.* [29,30,31] observed that QUIN in pathologic concentrations (0.1 and 1 mM) causes oligodendrocyte apoptosis. Neuronal, astroglial, and oligodendroglial cell death has been described on chronic QUIN exposure. Pierozan *et al.* [32] revealed that QUIN changes the structures of various proteins (tau, neurofilaments, *etc.*), resulting in neuron degeneration.

### 4.2. Clinical Results

**Figure 1:**
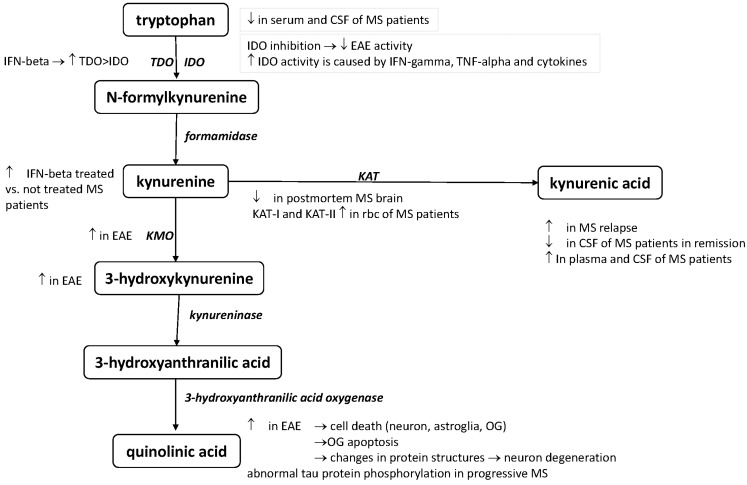
Alterations of the KP in EAE and MS. Alterations in the kynurenine pathway in EAE and MS—this schematic picture summarizes the alterations of the different kynurenine metabolites and enzymes in the animal model and human disease based on the presently published data. For list of abbreviations see page 9.

The evidence of subjects with MS found by Monaco *et al.* [33,34,35] that the Trp levels are lower in the serum and CSF corresponds with more recent results regarding depressed TRP levels in the serum and CSF of MS patients, proving the activation of KP in MS. An enhanced level of L-KYN was found in IFN-β treated MS patients relative to untreated RRMS patients [36]. Anderson *et al.* [37] identified a possible new role for QUIN, since they observed abnormal tau phosphorylation in progressive MS. Moreover, IDO-1 plays a very important role in regulating the immune response. During MS progression, it is very likely that the levels of pro-inflammatory cytokines such as IFN-γ and TNF-α increase in MS patients, thereby leading to IDO-1 and KP activation [38]. A number of research groups have found that many proinflammatory cytokines can activate IDO-1 [39,40,41]. Evidence has been presented that IFN-β can activate both pathways of the kynurenine cascade in the plasma of MS patients, and that IFN-β also affects IDO-1 (though to a lesser extent), which in turn decreases QUIN production [42]. In 2005, Hartai *et al.* [43] reported that serum levels of the KAT I and KAT II were significantly higher in the red blood cells of MS patients than in controls. Furthermore, the concentration of KYNA was found to be elevated in the plasma of MS patients; the same group also described the possible neuroprotective effect of KYNA in MS [43]. Elevated KYNA levels were described in the CSF of MS patiens [44]. The opposite was found in postmortem MS brain sections, with decreases in the concentrations of both enzymes responsible for KYNA production [45]. A low KYNA serum level was measured in the CSF of MS patiens in the remission [46]. Interestingly, elevated levels were found in acute relapse [47]. These controversial results may have originated from the disease groups (relapsing versus progressive) not being homogeneous at the time of sampling among the cited studies, e.g., kynurenines have been measured in different phases of the disease [47]. These findings point to the possible preventing role of KYNA in the acute phase of MS. The lower KYNA levels seen in the progressive phase of the disease shift the KP to neurotoxicity. Alterations of the KP have been revealed in all phases of the disease. To summarize the above results, KYNA is involved in possible neurotoxic processes as a protective agent, underlining its importance in neurodegenerative mechanisms (Figure 1, Table 1).

**Table 1:** The role of kynurenine pathway in MS and its experimental animal model-findings in connection with the disease.

Alterations of the KP Metabolism	References
Elevated serum and CSF TRP levels in MS patients with acute relapse	[33]
Depressed serum and CSF TRP levels in MS	[34,35]
KAT I and KAT II serum levels were significantly higher in red blood cells of MS patients	[43]
Decrease the concentrations of KAT I and KAT II enzymes in postmortem MS brain sections	[45]
KYNA concentrations elevated in the plasma of MS patients	[43]
Elevated KYNA levels in the cerebrospinal fluid of MS patients	[44]
Low KYNA serum level in the CSF of MS patients in remission	[46]
Elevated KYNA level in the CSF with acute relapse	[47]
3-HK is increased in EAE rats	[27]
KMO enzyme immunoreactivity has been found in cytoplasmic granules in the spinal cord and brainstem of rats with EAE	[27]
QUIN level increased in the spinal cords of EAE rats	[28]

KMO: kynurenine 3-mono-oxygenase; QUIN: quinolinic acid; EAE: experimental autoimmune encephalomyelitis; KAT I-II: kynurenine aminotransferase-I and II; KYNA: kynurenic acid; CSF: cerebrospinal fluid; TRP: tryptophan; 3-HK: 3-hydroxykynurenine; MS: multiple sclerosis.

## 5. Possible Therapeutic Targets Related to the KP

**Figure 2:**
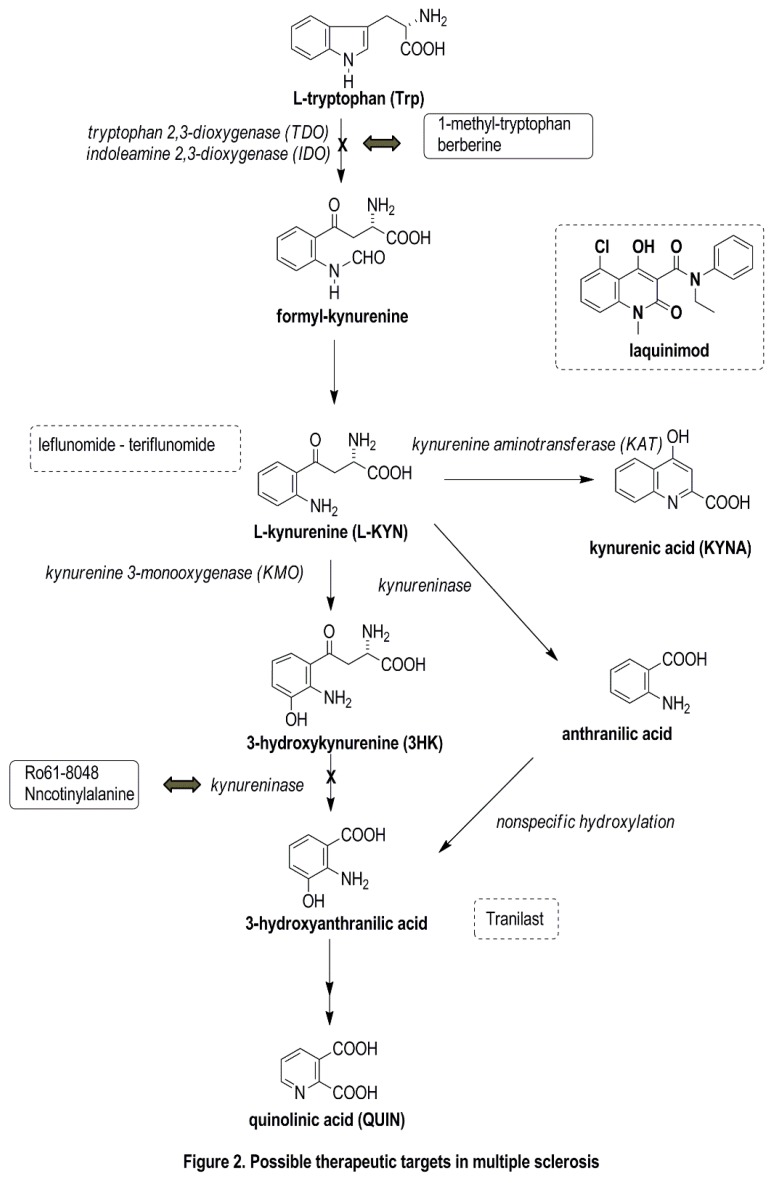
Possible therapeutic targets in MS. Possible therapeutic targets in the kynurenine pathway—the figure displays the future drug candidates which are influencing the kynurenine pathway (molecules indicated in the frames) or have structural similarities (represented in dashed line boxes) with kynurenine metabolites.

The treatments currently available for MS are all anti-inflammatory as concerns the mechanism of their effects, reducing the number and duration of attacks. At the present time, no neuroprotective molecules that would facilitate remyelination are available. An NMDA receptor antagonist was reported to modify the course of EAE and to prevent blood-brain barrier breakdown [48]. The NMDA antagonist KYNA and its pharmacological derivatives, together with KP enzyme inhibitors, might be promising new drugs that could fill the therapeutic gap (Figure 2). KYNA, a neuroprotective TRP metabolite, is itself not able to cross the blood-brain barrier; passage is possible by the ester form or by KYNA analogs [49]. One possible mode of beneficial therapeutic intervention is to shift the KP metabolism toward the formation of protective agents. This metabolic shift can be achieved by specific inhibitors of KMO, kynureninase and 3-hydroxi-anthranilic acid dioxygenase. A considerable number of possible pharmaceuticals have been developed with the aim of inhibiting these enzymes [50,51,52]. The systematic use of Ro 61-8048 as a selective KMO inhibitor led to accumulation of the neuroprotective KYNA and to decreases in the elevation of both 3HK and QUIN in the rat spinal cord [27]. An orally active synthetic Trp metabolite (*N*-[3,4-dimethoxycinnamoyl]-anthranilic acid (3,4-DAA), also known as Tranilas) has been examined in both *in vitro* and *in vivo* experiments (in microglial cells and in the RR version of EAE). Platten *et al.* [53] showed that this synthetic Trp metabolite decreased the proliferation of myelin-specific T cells and inhibited the proinflammatory cytokines produced by TH1 cells. The animals treated with 3,4-DAA suffered fewer and milder relapses, and there were decreases in the number of inflammatory nodes in the brains and spinal cords of the treated mice, verifying the immunosuppressive effect of this molecule, and indicating its possible use in TH1-mediated autoimmune diseases, e.g., MS. Laquinimod, a quinoline carboxamide, displays structural similarities with the Trp metabolites 3-HAA, 3-HKA and KYNA, and it might cross the blood-brain barrier [54,55,56]. One study revealed the role of laquinimod in immunoregulation, as it proved to decrease the antigen presentation and T-cell chemotaxis in APCs, and to shift the immune response from Th1 to Th2 and to affect Treg cells [57,58,59,60,61]. Similarly to glatiramer acetate, laquinimod treatment promoted the development of regulatory monocytes, revealing an increased secretion of IL-10. This made it capable of inhibiting the secretion of the proinflammatory cytokine IFN-γ [62]. Besides shifting the cytokine responses toward Th2, it prevented the proliferation of autoreactive T cells in the CNS. In a phase 3 study, laquinimod decreased the progression of the damage, and also the annual relapse rate to a lesser extent [63].

Sundaram *et al.* [64] inhibited the production of IDO-1 in a BV2 microglial cell culture, which in turn decreased QUIN production. IDO-1 inhibitors (1-MT and berberine) at 4 μM concentration significantly affected the QUIN levels and fully eliminated the oligodendrocyte apoptosis. The use of KP inhibitors could be a new strategy in the treatment of MS.

Cinnabarinic acid is an endogenous tryptophan metabolite of the KP, and a partial agonist of the type-4 metabotropic glutamate receptor (mGluR4), which proved to be protective in EAE [65]. Fallarino *et al.* [66] observed Th17 cell differentiation in the peripheral dendritic cells of mGluR4 knockout EAE rodents after initiation of the immune response. The expression of mGluR4 was found in wild-type EAE mice. After cinnabarinic acid administration, the expression of mGluR4 activation led to T cell differentiation in favor of the Th17 cells, which are responsible for immune tolerance and most probably protection against EAE. When Fazio *et al.* [67] treated mGlu4 knockout mice with cinnabarinic acid, the immune response was shifted toward T reg cell production. The latter offers a new therapeutic option in MS, by activating the KP pathway, changing the immunotolerance, and leading to a decrease in neuroinflammation.

An increase in interleukin-18 level, which activates the induction of IDO and QUIN, may partially mediate seizure activity in MS by elevating the IFN-γ level. Through this route, antiepileptic drugs might modulate wider MS symptomatology, interfering with melatonin and vitamin D3 [68].

## 6. Conclusions

Several endogenous neuroprotective mechanisms have recently been identified in MS, involving protective autoimmunity, direct low molecular weight antioxidants, indirect antioxidants inducing cytoprotective proteins, kynurenines, ischemic preconditioning, an integrated cell response, cannabinoids and the complement system. The result depends on the microenvironmental system [69]. The KP is a promising novel target through which to influence the immune responses and to achieve neuroprotection. Further research is therefore needed with the aim of developing novel therapeutics in MS and other autoimmune diseases.

## List of Abbreviations

3HK: 3-hydroxi-kynurenine
AMPA: α-amino-3-hydroxy-5-methyl-4-isoxazolepropionic acid
CSF: cerebrospinal fluid
EAE: experimental autoimmune encephalomyelitis
IDO: indoleamine-2,3-dioxygenase
IFN-β: interferon-beta
IFN-γ: interferon-gamma
KAT: kynurenine-aminotransferase
KMO: kynurenine-3-monooxygenase
KP: kynurenine pathway
KYNA: kynurenic acid
L-KYN: L-kynurenine
MS: multiple sclerosis
MRI: magnetic resonance imaging
NMDA: *N*-methyl-d-aspartate
NO: nitrogen-monoxid
OG: oligodendrocyte
QUIN: quinolinic acid
RRMS: relapsing-remitting multiple sclerosis
TDO: tryptophane-2,3-dioxygenase
TNF-α: tumor necrosis factor-alpha
Trp: tryptophan
